# Update on Pulmonary Langerhans Cell Histiocytosis

**DOI:** 10.3389/fmed.2020.582581

**Published:** 2021-03-08

**Authors:** Elzbieta Radzikowska

**Affiliations:** III Department of Lung Diseases and Oncology, National Tuberculosis and Lung Diseases Research Institute, Warsaw, Poland

**Keywords:** pulmonary Langerhans cell histiocytosis, adults, lung, BRAF, MAPK

## Abstract

Pulmonary Langerhans cell (LC) histiocytosis (PLCH) has unknown cause and is a rare neoplastic disorder characterized by the infiltration of lungs and various organs by bone marrow-derived Langerhans cells with an accompanying strong inflammatory response. These cells carry somatic mutations of *BRAF* gene and/or *NRAS, KRAS*, and *MAP2K1* genes, which cause activation of the mitogen-activated protein kinase (MAPK)/extracellular signal-regulated kinase (ERK) signaling pathway. PLCH occurs predominantly in young smokers, without gender predominance. Lungs might be involved as an isolated organ or as part of a multiorgan disease. High-resolution computed chest tomography plays an outstanding role in PLCH diagnosis. The typical radiological picture of PLCH is the presence of small intralobular nodules, “tree in bud” opacities, cavitated nodules, and thin- and thick-walled cysts, frequently confluent. Histological examination of the lesion and demonstration of characteristic eosinophilic granulomas with the presence of LCs that display antigen CD1a or CD207 in immunohistochemistry are required for definite diagnosis. Smoking cessation is the most important recommendation for PLCH patients, but treatment of progressive PLCH and multisystem disease is based on chemotherapy. Recently, new targeted therapies have been implemented.

## Introduction

Pulmonary Langerhans cell (LC) histiocytosis (PLCH) is a rare neoplastic disorder of unknown etiology, characterized by the infiltration of the lungs and various organs by bone marrow-derived LCs with an accompanying strong inflammatory response (1). These cells carry somatic mutations of the *BRAF* gene and/or *NRAS, KRAS*, and *MAP2K1* genes, which cause activation of the mitogen-activated protein kinase (MAPK)/extracellular signal-regulated kinase (ERK) signaling pathway (2–5).

The histiocytic diseases have been reclassified into five categories; LCH is considered a member of group L, along with Erdheim–Chester disease, indeterminate cell histiocytosis, and mixed LCH/Erdheim–Chester disease (1, 6). PLCH occurs predominantly in young smokers and shows no predominance in either sex. The lungs may be involved as isolated organs or as part of a multisystem disease (7). Proliferating LCs form nodular lesions of various sizes, which infiltrate neighboring tissues and damage their structure. In adults, LCH most commonly affects the lungs, bones, skin, and pituitary gland. The involvement of lymphopoietic organs (e.g., lymph nodes, liver, spleen, and bone marrow), alimentary system, and central nervous system (CNS) is rare. In the course of LCH, pulmonary lesions may be isolated, occur after chronic systemic disease, or are an initial sign of disease. The isolated pulmonary form of LCH is observed in approximately 50–70% of patients with PLCH (8–11).

***Single-system LCH (SS-LSH)*** comprises single or multiple involvement of the following single organs or systems:
***Bone*,** with involvement of a single or multiple bones and many foci in many bones;
***Skin***;
***Lymph nodes***, excluding lymph nodes that drain the area of histiocyte infiltration or multiple lymph nodes (i.e., more than one lymph node group);
***Hypothalamus–hypophysis/CNS***;
***Isolated pulmonary involvement***;
***Other***, with involvement of oral mucosa, thyroid, thymus, or intestine.
***Multisystem LCH (MS-LCH)*** comprises disease in which two or more organs or systems are affected:
***Involvement of critical organs***, e.g., hemopoietic system, spleen, liver;
***No involvement of critical organs*.**

The involvement of specific areas and bones [e.g., vertebrae (possible compression spinal fracture and damage to the spinal cord), orbital bones, mastoid process, sphenoid bone, and temporal bones with transition into soft tissue (possible injury to facial nerves and pituitary gland)] is associated with the risk of CNS involvement, which impacts decisions regarding treatment (7, 12).

Previously, the lungs were considered as a “risk organ,” but multivariable analysis of cohorts of 420 children with MS-LCH showed that lung involvement was not the significant prognostic variable (13). However, patients with lung lesions have a high risk of developing life-threatening complications (e.g., pneumothoraces, pulmonary infections). Recently, Le Louet et al. (14) presented the assessment of French LCH registry, and it was found that severe lung involvement in children was associated with high mortality. These findings suggested revision of treatment guidelines for this group of patients.

## Epidemiology

LCH has an estimated prevalence in adults of 1–2/1,000,000. However, there have been few population-based epidemiological studies regarding this disease. In Japanese hospitalized patients, the prevalence rates of PLCH were reported to be 0.07/100,000 in women and 0.27/100,000 in men. PLCH is presumed to affect approximately 5% of patients undergoing open lung biopsy. The prevalence of PLCH may be underestimated due to the nature of the disease (7, 15).

PLCH affects young people in the third and fourth decades of life, with no sex predominance. Approximately 90 to 95% of patients with PLCH are tobacco smokers (16, 17). Recently, it was noticed that 20–33% of patients smoked both cigarettes and cannabis [(18), personal observations]. The intensity and smoking duration do not influence the development of PLCH. Isolated PLCH in children is extremely rare; the disease is reportedly associated with passive exposure to tobacco smoke in the majority of pediatric patients, and ~10–30% of children with multisystem LCH exhibit pulmonary lesions (11, 19). Familial occurrence of LCH has been reported, but no genetic predisposition toward LCH has been found (7).

## Pathogenesis

There have been many investigations regarding the pathogenesis of LCH; the finding that dendritic cells with mutations in MAPK pathway genes contribute to the development of the disease has yielded new insights. Dendritic cells are a heterogeneous group of cells, which mainly serve to process and present antigens to immune cells. Normal LCs are present in the skin, as well as beneath the epithelia of the bronchial tree and other mucosae (3, 6). Toll-like receptors, expressed on pathogen-sensing cells, and factors released from damaged or dying cells, stimulate the activity of normal LCs. Subsequently, the immunological response involves the migration of activated dendritic cells to the nearest lymph nodes and presentation of antigens to naive T lymphocytes. In addition, tolerance to harmless inhaled antigens is mediated by normal LCs (20).

Tobacco smoke plays a major role in the development of PLCH (21). It causes inflammatory cell accumulation in the lungs; these cells include LCs, which release cytokines such as tumor necrosis factor alpha, interleukin 1 beta, granulocyte-macrophage colony-stimulating factor, transforming growth factor beta, and the dendritic cell chemokine (chemokine ligand 20) (22). In addition, bronchial epithelial cells and fibroblasts release granulocyte-macrophage colony-stimulating factor, which is a strong mitogenic factor for LCs.

Moreover, PLCH lesions exhibit elevated expression of osteopontin, a glycoprotein that induces chemotactic activity in macrophages, monocytes, and dendritic cells, including LCs. Activated pathological LCs show strong lymphocyte-stimulating properties and are characterized by elevated expression of CD40, CD80, and CD86. Previous studies have yielded inconsistent results regarding the importance of interleukin-17 released by T lymphocytes in the pathogenesis of histiocytic lesions. This cytokine promotes the development of giant cells and formation of granulomas. Elevated expression of the antiapoptotic protein, Bcl-xL, within histiocytic granulomas maintains the pathogenic process (23).

In addition to inflammation, granuloma formation is accompanied mPAP, mean pulmonary arterial presure; by remodeling of the lung parenchyma. Cystic destruction of the lung is presumably caused by activation of metalloproteinases 2 and 9, produced by dendritic cells, LCs, and monocytes. Moreover, elevated expression of transforming growth factor beta released by granuloma cells causes fibrotic lesions (12).

There have been a number of investigations regarding the pathogenesis of PLCH. Inflammatory cell accumulation and the presence of LCs with dysfunctional apoptosis, as well as clonal proliferation, have been associated with the onset of PLCH.

**Figure 1:**
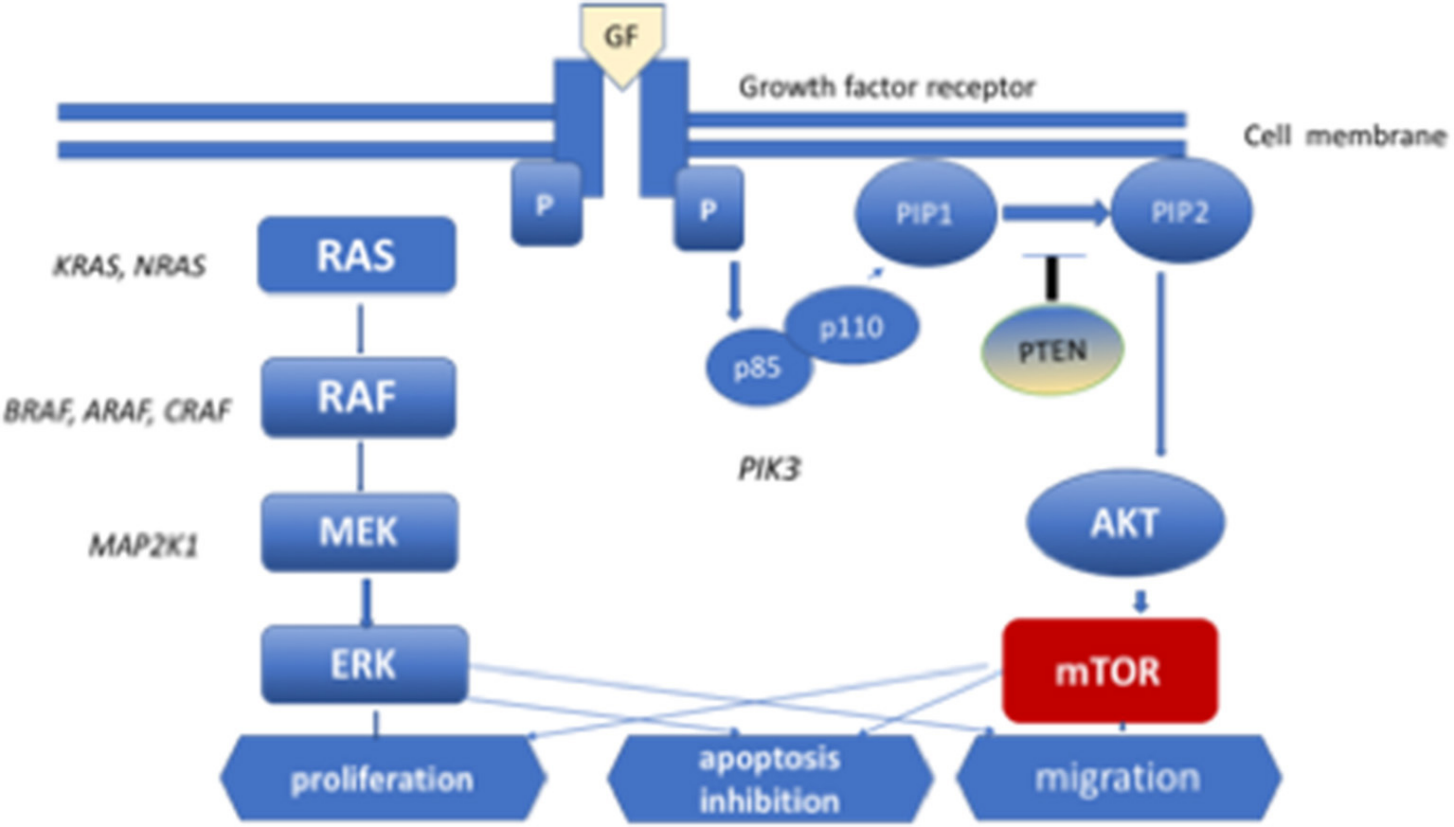
MEK-mitogen activated protein kinase–extracellular signal-regulated kinase (ERK) signaling cascade of the mitogen-activated protein kinase (MAPK) pathway and phosphoinositide 3-kinase (PI3K)–AKT-serine/threonine protein kinase Akt pathway. Diagram of RAS protein activation.

Identification of the role of the RAS/MAPK signaling pathway in the pathogenesis of LCH was a key discovery regarding the pathogenetic mechanism of this disorder (24). This pathway transmits proliferative signals from the cell surface *via* the RAS pathway to phosphorylate MAPK and ERK, which transmit signals to the nucleus. The MAPK pathway regulates the activities of many enzymatic proteins and transcription factors. Activating mutations of the BRAF, ARAF, MAP2K1, N/K/HRAS, and PIK3CA genes have been found in patients with PLCH (25–27) (Figure 1). The *BRAF* V 600E mutations were found in 50% of LCH pulmonary nodules and MAP2K1 in an additional 20% of cases (26). Moreover, whole-exome sequencing analysis revealed *MAP2K1* mutations in seven of 21 *BRAF*-wild-type LCH biopsy tissue samples, but not in *BRAF*-mutant specimens (5). The activation of ERK was detected in >90% of patients with LCH. In patients with multisystem LCH, mutation of the *BRAF* V 600E gene may occur in myeloid cells; in patients with single-system LCH, mutations may occur in cells originating from peripheral lesions (28). The single-cell analysis showed cellular, transcriptomic, and epigenomic heterogeneity among LCH cells, which ware connected with the development of LCH lesions. LCH cells share enrichment of genes involved in interferon (IFN) signaling and antigen presentation, indicating the inflammatory nature of the disease. In addition, genes involved in cell cycle pathways and DNA repair were presented. It is suggested that interindividual differences in subsets of LCH cells may be connected with clinical presentation of the disease. Validation of the differences in LCH subset composition in bulk RNA sequencing, immunohistochemistry, immunofluorescence imaging in a large cohort of LCH patients might be helpful in patient management (29).

Mutations in genes involved in the MAPK pathway related to precursors of distinct macrophage types (i.e., bone marrow or yolk sac) result in the occurrence of an overlapping syndrome of LCH and Erdheim–Chester disease or chronic myeloid leukemia. Notably, PLCH was recently recognized as a neoplasm with a strong inflammatory component (30).

## Clinical Presentation

### Respiratory Symptoms

Patients with PLCH exhibit dry cough (50–70%), reduced exercise tolerance (40–80%), exertional dyspnea (40–87%), fatigue (50–80%), weight loss (20–30%), chest pain (10–30%), night sweats (10–20%), and fever (10–15%). Approximately 10–30% of affected patients are diagnosed following incidental pneumothorax. Pneumothorax occurs during the course of disease in 30–45% of affected patients (17, 31). Changes are found incidentally on routine chest X-rays without symptoms in 5–25% of affected patients. Dyspnea at rest and characteristics of right-ventricular circulatory failure occur in the late stages of PLCH. More than 10% of patients with PLCH develop pulmonary hypertension; it is not always related to the exacerbation of pulmonary lesions but may be due to the involvement of pulmonary vessels that occurs during the course of the disease (9, 11). Pulmonary hypertension and dynamic hyperinflation are the most important factors that limit exercise capacity in PLCH patients (32). Moderate or severe pulmonary hypertension [mean pulmonary arterial pressure (mPAP) >35 mmHg] was revealed in 92% of the PLCH patients who presented for lung transplantation (33).

Between 25 and 50% of patients without pulmonary symptoms at the time of presentation show symptoms related to other organ involvement, such as polyuria–polydipsia (20–30%), bone pain (20–50%), and oral mucosa or skin lesions (~10%). Initial evaluation of patients with LCH requires whole-body assessment. Symptoms typically arise 6–20 months prior to recognition of the disorder; in some affected patients, the diagnosis is established after many years of observation. Patients with spontaneous pneumothorax as an initial symptom of the disease are reportedly younger, more frequently men, and exhibit greater respiratory impairment compared with those who do not have pneumothorax (17).

### Radiological Findings

#### Chest Radiography

**Figure 2:**
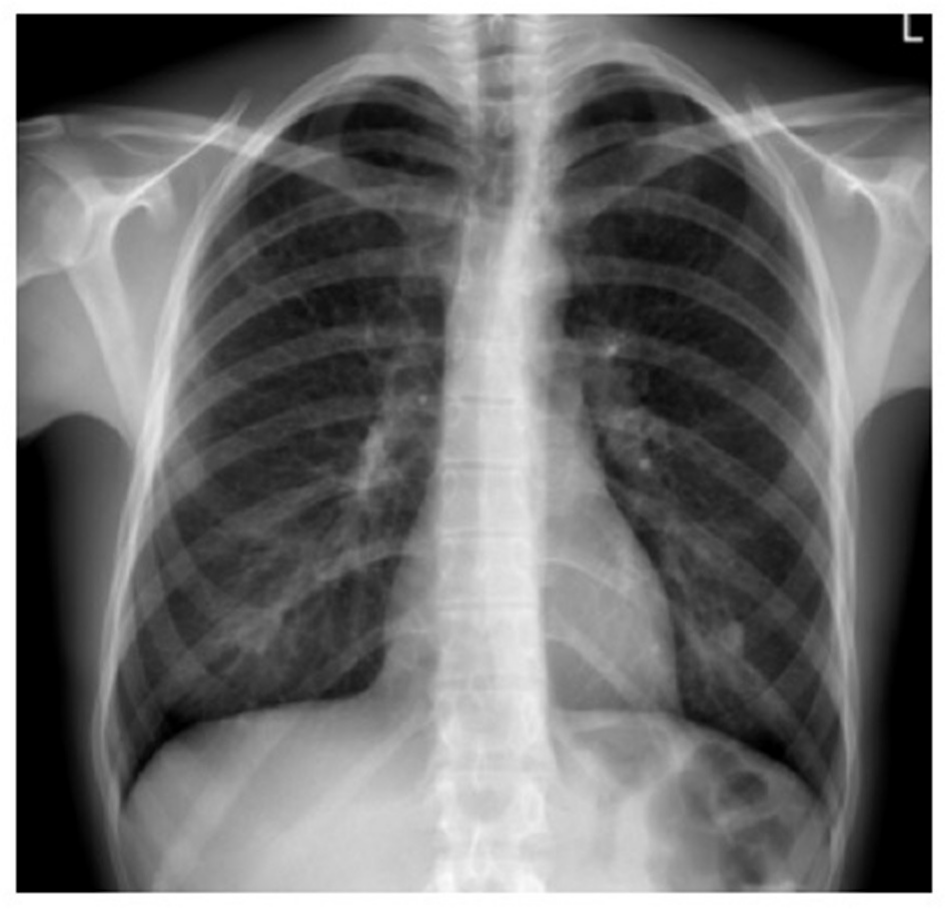
X-ray film of a pulmonary Langerhans cell histiocytosis (PLCH) patient. Multiple reticular and nodular changes in the upper and middle parts of both lungs, with an increase in lung volume.

Standard chest radiography has limited value, as multiple lesions may be small. In patients with advanced disease, nodular, reticular, and cystic lesions are visible in the middle and upper lung fields (Figure 2). Lung volumes are typically preserved but may be diminished in patients with a history of pneumothorax and pleurodesis. Enlargement of the hilar and mediastinal lymph nodes is present in approximately 10% of affected patients (11).

#### Computed Tomography

**Figure 3:**
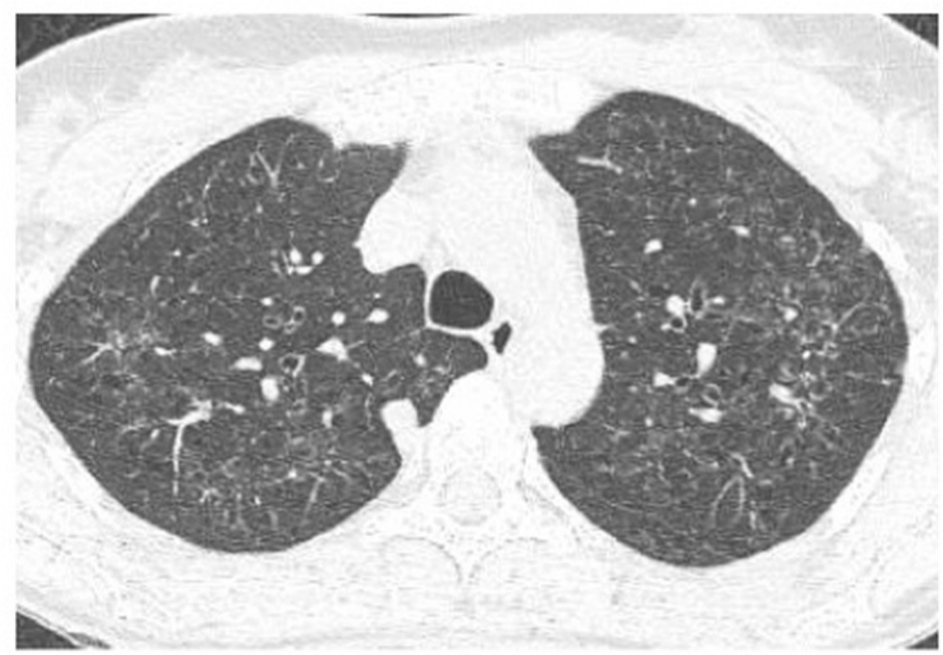
High-resolution computed tomography (HRCT) scan of a pulmonary Langerhans cell histiocytosis (PLCH) patient. Multiple intralobular nodules, nodules with cavitations, and small nodular lesions in both lungs.

**Figure 4:**
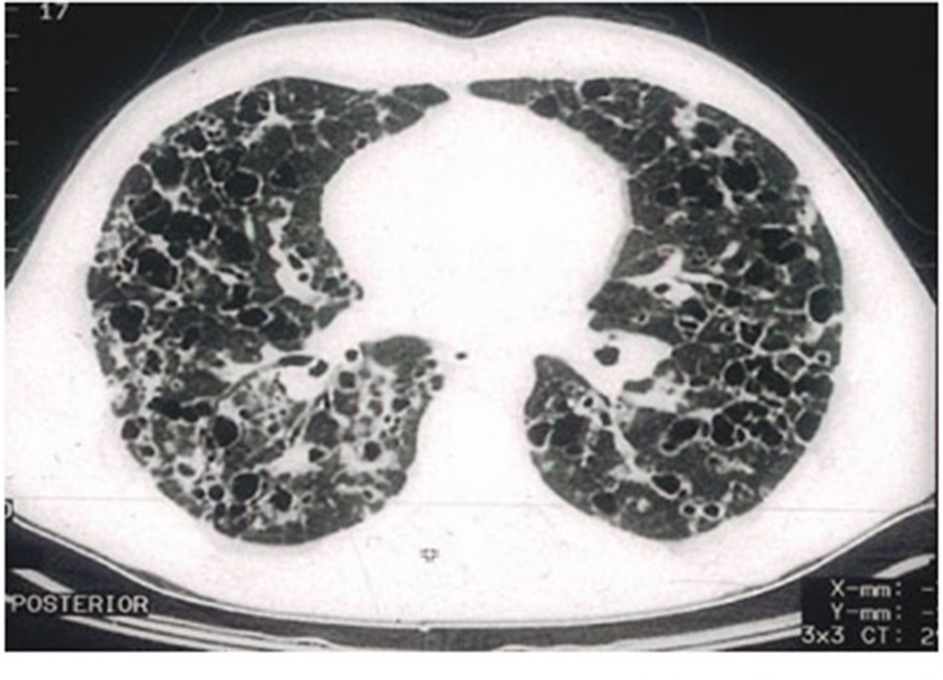
High-resolution computed tomography (HRCT) scan of a pulmonary Langerhans cell histiocytosis (PLCH) patient. Multiple cystic lesions, frequently confluent with bizarre shapes.

**Figure 5:**
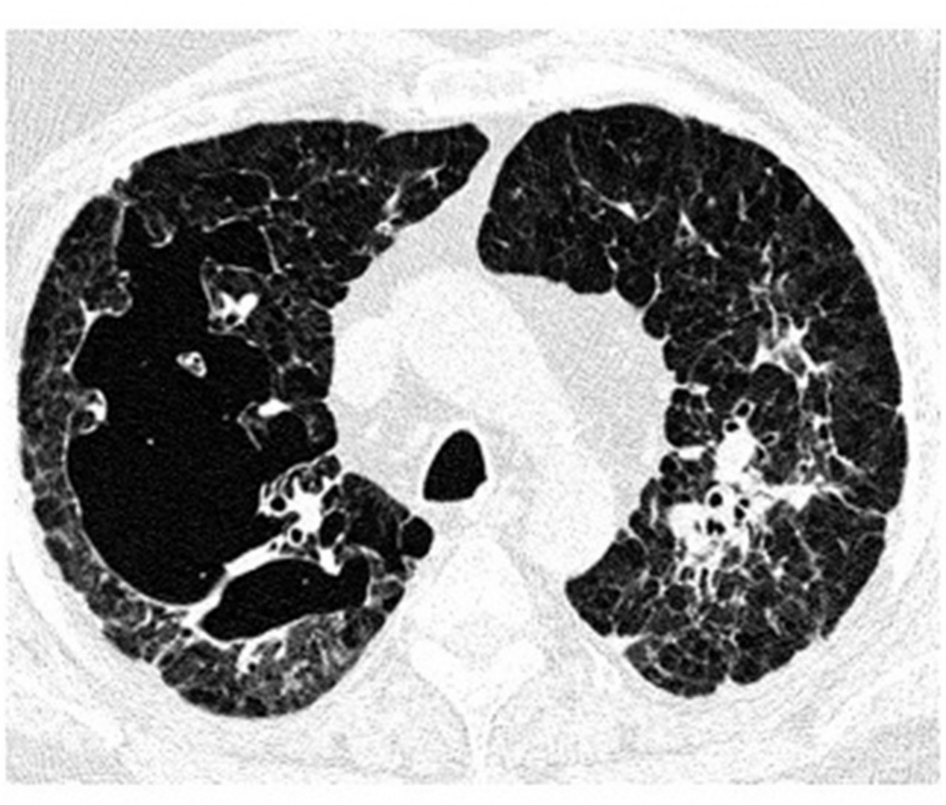
High-resolution computed tomography (HRCT) scan of a pulmonary Langerhans cell histiocytosis (PLCH) patient. Large confluent cystic lesions in both lungs.

High-resolution computed tomography (HRCT) plays a major role in the diagnosis of PLCH (7, 34). The typical findings are centrilobular nodules (frequently with a “tree-in-bud” appearance), nodules with or without a lacuna, and initially thick-walled cysts of various shapes (these may be isolated or confluent with a “cloverleaf” appearance) (Figures 3–5). As the disease progresses, the cysts become larger and thin-walled. Lesions have a characteristic distribution, with predominance in the upper and middle parts of the lungs; the costophrenic angles are spared (>90%). In children, both lungs are symmetrically affected and lesions may be present in the lower lobes (14). In some patients, different degrees of pneumothorax are present. In patients with PLCH, other radiological symptoms of smoking-related diseases are often visible (e.g., emphysematous bullae or ground-glass opacities). Enlarged lymph nodes may be present in approximately 10% of affected patients. Signs of pulmonary hypertension, megalocardia, and enlarged pulmonary trunk are observed in patients with advanced disease (35).

#### Positron Emission Tomography

Positron emission tomography with ^18^F-fluorodeoxyglucose has limited value in the assessment of patients with PLCH. Only 20–25% of patients show higher ^18^F-fluorodeoxyglucose uptake in the lungs, particularly in thick-walled cysts and nodular lesions. Positron emission tomography is more sensitive than CT in terms of identifying bone lesions, particularly those that are subclinical, as well as those that involve lymph nodes, liver, spleen, or thyroid. Positron emission tomography is reportedly sensitive for the assessment of early response to treatment and disease relapse (36, 37).

#### Laboratory Tests

The results of laboratory tests are usually unremarkable. However, patients with PLCH often show elevated levels of serum inflammatory markers. Serum hyperosmolarity with lower urine specific gravity and hypoosmolarity are observed in patients with diabetes insipidus. Elevated serum levels of hepatic enzymes and bilirubin are characteristic of liver involvement (7). Fluid phase biopsy is valuable for the assessment of *BRAF* mutations in patients who are potential candidates for targeted therapy.

#### Pulmonary Function Testing

Patients with PLCH exhibit various patterns of ventilation disorders. Initially, approximately 20% of affected patients have normal values on pulmonary function tests. Obstruction with hyperinflation is the main abnormality and can often be reversed to some degree. Restricted breathing is uncommon and mainly affects individuals with recurrent pneumothorax and pleurodesis. The most common abnormality is reduced diffusing capacity of the lungs for carbon monoxide, which is observed in approximately 70–90% of affected patients. The 6-min walk test is a useful examination; desaturation during exercise is a sensitive marker of lung impairment in patients with less advanced disease, and a reduced walking distance is observed in patients with advanced disease (17, 38, 39).

#### Bronchoscopy and Bronchoalveolar Lavage

Bronchoscopy is typically performed to exclude other disorders, especially infections. The diagnostic value of transbronchial biopsy is limited and estimated at 10–50% due to focal distribution of lesions, despite the bronchiolocentric nature of the disease (40, 41). Transbronchial biopsy is most useful in patients with nodular lesions but in patients with cystic lesions is associated with a high risk of pneumothorax. A new technique for lung tissue sampling, cryobiopsy, yields a diagnosis in ~70% of patients with interstitial lung disease. The value of this technic in PLCH is under evaluation because in only a few cases has diagnosis of PLCH been established by cryobiopsy (42, 43). The presence of >5% cells with CD1a expression in bronchoalveolar lavage (BAL) fluid with a typical radiological pattern on chest HRCT is specific for PLCH but is detected in only 0–25% of affected patients (44). Auerswald et al. (45) noticed that all their six patients with histologically proven histiocytosis displayed more than 5% CD1-positive cells, but Torre and Harari (46) were able to establish PLCH in four (25%) out of 16 patients on the basis of BAL assessment. Similarly, only three (38%) out of eight patients with PLCH presented by Baqir et al. (41) and 10 (25%) out of 40 patients presented by Elia et al. (11) in whom BAL was performed displayed the presence of CD1a-positive cells over 5%. In our group of 38 patients with PLCH, only in eight (21%) did BAL assessment have a diagnostic value (47).

#### Lung Biopsy

Open lung biopsy is the gold standard for a definitive diagnosis of PLCH. In patients with LCH confirmed by histological examination of specimens obtained from other foci (e.g., skin, mucosa, or bones), it is not necessary to confirm pulmonary lesions (7).

#### Histological Examination

PLCH has a focal, bronchiolocentric localization. Initially, inflammation with eosinophilic granuloma formation occurs near small bronchi and bronchioles, destroying their walls; it exhibits varying degrees of extension into the adjacent lung interstitium. Frequently, macrophages with dark inclusions are observed; these macrophages are characteristic of exposure to inhaled fumes, mainly tobacco smoke. Nodules contain a mixture of inflammatory cells, such as T cells, macrophages, monocytes, and LCs; these LCs are relatively large, with a pale, slightly eosinophilic cytoplasm and a convoluted nucleus that exhibits a longitudinal crease resembling a coffee bean. Electron microscopic examination shows pentalammelar structures (consisting of lectin) in the cytoplasm associated with the cell membrane; these so-called Birbeck granules are characteristic of LCs. These structures can also be identified by immunohistochemistry using antibodies against langerin (i.e., CD207). LCs show CD1a expression, which is necessary for diagnosis (24). The expression of S-100 protein is not specific but may be present. Inflammatory eosinophilic granulomas may be observed, depending on the stage of disease progression. The central part of the nodule has a lacuna, which is presumably a component of the remaining bronchiole lumen or the effect of cytokine and metalloproteinase-mediated destruction. Subsequently, the inflammation regresses; however, fibrosis develops in the form of stellar scars or confluent cystic cavities in the fibrous ring. At this stage, LCs are absent. In LCH lesions, LCs constitute 1–80% of cells (mean ~8%) (3). Lung specimens from patients with PLCH may also show signs of other smoking-related diseases, such as bronchiolitis, desquamative interstitial pneumonia, respiratory bronchiolitis with accompanying interstitial lung disease, or emphysema. In patients with advanced disease, the internal walls of both arteries and veins are thickened, resulting in pulmonary hypertension (48).

Patients who qualify for targeted therapy must be tested for mutations in the *BRAF, ARAS, NRAS, KRAS*, and *MAP2K* genes (5). Moreover, as a novel diagnostic technique for monitoring patients undergoing treatment with BRAF inhibitors, analysis of BRAF V600E gene mutations in cell-free DNA in serum has been implemented (49, 50).

## Diagnosis

***Definite diagnosis of PLCH*** requires adequate clinical presentation and the identification of LCs in biopsy specimens that exhibit either CD207 (langerin) or CD1a.

***Probable diagnosis of PLCH*** relies on clinical presentation confirmed radiologically (characteristic cysts and nodules mainly observed in upper and middle lung fields on chest CT). Histological verification of all extrapulmonary lesions is recommended (7).

### Differential Diagnosis

Disorders that should be taken into account while diagnosing the patients with cystic pulmonary lesions are presented in Table 1.

**Table 1:** Diseases with a cystic pattern of lung lesions.

• Pulmonary Langerhans cell histiocytosis
• Lymphangioleiomyomatosis (LAM)
◦ Sporadic LAM
◦ Tuberous sclerosis complex LAM
• Birt–Hogg–Dubé syndrome
• Erdheim–Chester disease
• Lymphatic disorders
◦ Lymphocytic interstitial pneumonia
◦ Light-chain deposition disease
◦ Amyloidosis ◦ Hyper-IgE syndrome
• Genetic disorders
◦ Ehlers–Danlos syndrome
◦ Neurofibromatosis
◦ Marfan syndrome
◦ Proteus syndrome
• Congenital pulmonary airway malformations
• Infections
◦ Respiratory papillomatosis
◦ Staphylococcal pneumonia
◦ *Pneumocystis jirovecii*
◦ Endemic fungal diseases (coccidiomycosis, paragonimiasis)
• Emphysema
• Alpha-1 antitrypsin deficiency
• Post-traumatic pseudocysts
• Pulmonary neoplasms
◦ Adenocarcinoma
◦ Lymphoma
◦ Mesenchymal hamartoma
◦ Pleuropulmonary blastoma
• Sarcomas
◦ Angio and osteosarcomas
◦ Synovial cell sarcoma
◦ Leiomyosarcoma
◦ Rhabdomyosarcoma
◦ Endometrial stromal sarcoma
◦ Wilms tumor
◦ Ewing sarcoma
• Metastatic tumors
◦ Adenocarcinoma of genitourinary and gastrointestinal tract
◦ Breast Cancer
◦ Cancers of the head and neck

In patients with nodular lesions, diseases such as neoplasms, sarcoidosis, hypersensitivity pneumonitis, and infections are in the scope of differential diagnosis (7).

## Treatment

**Figure 6:**
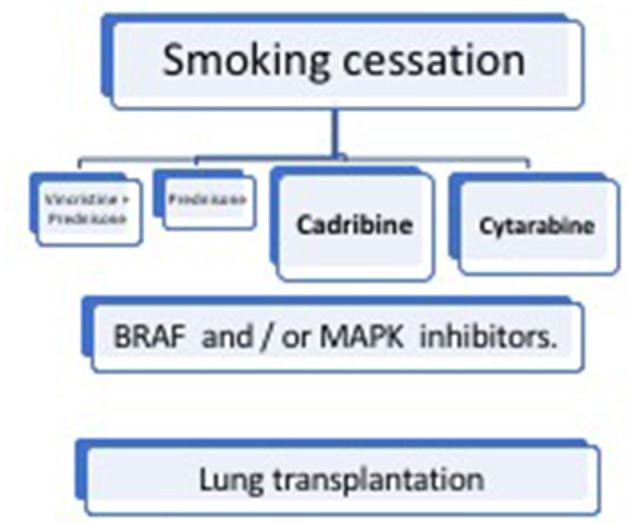
Management of pulmonary Langerhans cell histiocytosis (PLCH).

Treatment of LCH depends on the disease activity and affected organs, including lesions in critical organs and high-risk bones, as well as the degree of damage (Figure 6).

### Smoking Cessation

Because of the crucial role of tobacco smoke in the development of PLCH, smoking cessation is the most important recommendation for affected patients. In ~50% of patients with isolated PLCH, smoking cessation leads to partial regression and subsequent stabilization of the disorder without immunosuppressive therapy (16, 51). However, patients require systematic follow-up examinations because reactivation of the disease can occur in the lungs or other organs. Thus far, no biological markers have been found to identify patients in whom smoking cessation would be sufficient and patients in whom the disease is likely to progress despite smoking cessation. Cessation of both cigarette smoking and marijuana smoking is becoming a challenge for both patients and doctors [(52), personal observation].

### Glucocorticosteroids

Systemic corticosteroid therapy has been advised for many years; it has been reportedly associated with symptomatic, radiological, and functional improvements. However, there have been insufficient studies to determine the appropriate corticosteroid dose and duration of treatment, as well as comparative studies with respect to smoking cessation. It is possible that some effects of therapy are due to smoking cessation, rather than steroid treatment (10). In addition, relapse after treatment is common (~80%); long-term corticosteroid therapy is associated with many (sometimes severe) adverse effects. Therefore, this treatment is no longer recommended. However, inhaled corticosteroids may be useful for treatment of patients with reversible obstruction (7).

### Chemotherapy

Various chemotherapy regimens have been proposed for use in children with LCH, but they have failed to show satisfactory results in adults. The disease has a diverse clinical course in adults, particularly in patients with isolated PLCH, and many drugs are differently tolerated (53–55). Vinblastine, methotrexate, mercaptopurine, and etoposide have shown benefits in some patients with multisystem LCH, but their effects in patients with isolated PLCH are controversial (56). A retrospective study involving a population of 35 adults with LCH (17 patients with pulmonary involvement) treated with vinblastine and steroids showed a response rate of 70%; however, the relapse rate was 40% during the 5-year follow-up period (57). No improvements in ventilation parameters due to vinblastine treatment were observed in any patient. Cantu et al. (58) reported that vinblastine treatment was less effective than cladribine or cytarabine in adult patients with multifocal bone disease (28% had pulmonary lesions). However, the effects of these treatments on lung function parameters were not discussed. Recently, Néel et al. (59) reported an overall response rate of 91% in 22 patients treated with cladribine; however, disease progression occurred in 30% of these patients during the 5-year follow-up period.

Saven and Burian (60) reported that 2-chloro-2′-deoxyadenosine (2-CDA) had beneficial effects in 12 patients (six with lung involvement), affording an overall response rate of 75%. Grobost et al. (61) reported that cladribine (three or four courses) improved pulmonary function parameters in four of five patients with PLCH; however, one of these patients exhibited relapse. Pardanani et al. (62) and Adam et al. (63) in five and seven patients with PLCH who received 2-CDA, respectively reported over than 60% response rate.

Cladribine treatment in adult PLCH patients is a subject of an ongoing phase II clinical trial (NCT01473797, www.ClinicalTrials.gov). The most frequently observed severe adverse event during the course of cladribine treatment is cytopenia, whereas the most severe event associated with vinblastine is neuropathy. For patients with disease progression after cladribine treatment, salvage therapy is cytarabine administered at a dose of 100 mg/m^2^ on five consecutive days at 4-week intervals. Another phase II trial (NCT04121819, www.ClinicalTrials.gov) to investigate the efficacy and safety of cytarabine in adult patients with LCH is currently underway. Notably, the preferred chemotherapeutic regimen in patients with brain lesions is cytarabine and cladribine because both drugs cross the blood–brain barrier.

Clofarabine is another drug that is effective as salvage therapy in children and is the subject of another ongoing phase II clinical trial (NCT02425904). In addition, severe lung cystic lesions and chemotherapy, particularly with concomitant corticosteroid treatment, are predisposing factors to opportunistic infections, particularly *Pneumocystis jiroveci* pneumonia. Grobost et al. (61) recommended that sulfamethoxazole/trimethoprim and valaciclovir prophylaxis should be administered during cessation of 2-CDA treatment, as well as for 6 months afterward.

### Targeted Therapies

#### Vemurafenib and Other BRAF Inhibitors

The presence of mutations in genes involved in the MAPK pathways in patients with PLCH implies that targeted therapy may be useful. Vemurafenib, a BRAF kinase inhibitor, is reportedly an effective treatment in patients with LCH (64, 65, 70). However, this drug did not eliminate all LCs; discontinuation of treatment resulted in disease progression. Diamond et al. (67) reported the beneficial effects of vemurafenib in a group of 22 patients with Erdheim–Chester disease and four patients with LCH; these patients exhibited a 2-year progression-free survival rate of 86% and an overall survival rate of 96%. Notably, the patients with LCH showed a particularly good response. Bhatia et al. (68) reported a partial response in patients with refractory Erdheim–Chester disease overlapping with LCH during treatment with another BRAF inhibitor, dabrafenib. Recently, Hazim et al. (66) presented results of treatment with vemurafenib and dabrafenib in six adult patients with LCH. Complete and partial responses were noticed in 30 and 50% of patients, respectively (Table 2).

**Table 2:** Targeted therapies in LCH adult patients.

**Study**	**Trial**	**Patients**	**BRAF inhibitor**	**Time of response assessment**	**Response**
Hyman (70)	Phase 2	13 ECD 1 LCH	Vemurafenib	2 months	ORR-43%
Diamond et al. (67)	Phase 2	22 ECD 4 LCH	Vemurafenib	2–40 months	ORR-61%
Bhatia (68)	Retrospective	7 ECD 4 ECD/LCH	Dabrafenib	2–40 months	ORR-100%
Diamond et al. (69)	Prospective	12 ECD 2 LCH	Cobimetinib	12 months	ORR-89%
Hazim (66)	Retrospective	6 LCH	3 Vemurafenib 3 Dabrafenib	4–27 months	CR-33% PR-50%

*CR, complete response; ECD, Erdheim–Chester disease; LCH, Langerhans cell histiocytosis; ORR, overall response rate; PR, partial response*.

In a study of children with LCH performed in France, the presence of *BRAF* mutations was associated with a weaker response to first-line treatment (vinblastine and steroids) and second-line treatment, as well as a higher proportion of recurrence (65, 71). However, no correlations were found between *BRAF* mutations, clinical presentation, and prognosis in adults (5).

#### Mitogen-Activated Protein Kinase Inhibitors

Drugs that inhibit mitogen-activated kinase 1 (MEK1) and 2, both downstream of BRAF, were shown to be beneficial in patients with LCH who exhibited *MAP2K1* deletion. Lorillion et al. (72) reported a good response to trametinib. Cobimetinib, a MEK1 and 2 inhibitor, was the subject of a clinical trial in patients with various histiocytic disorders. All patients showed a response to this treatment; the relapse rate was only 10% at 1 year (69). Studies regarding melanoma cells harboring mutations in genes involved in the MAPK pathway showed that dual inhibition of BRAF and MEK was more effective and less toxic than monotherapy with a BRAF inhibitor. Awada et al. (73) reported beneficial results of this treatment in adult patients with multisystem LCH. Multiple clinical trials involving BRAF and other RAS pathway inhibitors in adults and children with LCH are currently underway (www.ClinicalTrials.gov).

#### Tyrosine Kinase Inhibitors

Contradictory results have been reported regarding the effects of imatinib treatment. Montella et al. (74) and Janku et al. (75) reported beneficial effects of this drug in a 37-year-old woman with multisystem LCH and in two adult patients with multisystem LCH, respectively. However, the follow-up periods in these patients were both shorter than 2 years. In contrast, Wagner et al. (76) reported treatment failure in two patients treated with imatinib.

#### Pneumothorax

Pneumothorax, as the first symptom of PLCH, is observed in 10–30% of affected patients. In addition, these patients have a greater probability of pneumothorax recurrence (~60%). Generally, 30–40% of patients develop this condition during long-term observation. Moreover, this group often includes young men who smoke fewer cigarettes and whose lung function is more affected by the disease. These data support the recommendation that HRCT should be performed in all patients with spontaneous pneumothorax to identify patients with possible PLCH (17, 31, 77).

Pleurodesis should be recommended after the first episode of pneumothorax. However, it was recently reported that the time of the first ipsilateral recurrence and the overall number of pneumothorax recurrences were similar after conservative and thoracic surgical treatments. Pneumothorax recurrences may be linked with active disease (18). Notably, pleurodesis does not constitute a contraindication for lung transplantation (7).

#### Treatment of Pulmonary Hypertension

The progression of PLCH leads to the destruction of lung parenchyma with elements of fibrosis, as well as the development of pulmonary hypertension. In a small proportion of patients, those with stable ventilation parameters may exhibit pulmonary hypertension.

Oxygen treatment is the main recommendation for this group of patients. Furthermore, drugs that lower pulmonary artery blood pressure (e.g., inhibitors of phosphodiesterase and endothelin receptor, as well as prostacyclin) were shown to have beneficial effects in patients with PLCH who exhibited pulmonary hypertension (78). Also other small series of patients and case reports presented beneficial effects of pulmonary antihypertensive therapies; however, it is not proven as standard treatment and should be delivered in very experienced centers (79, 80).

#### Lung Transplantation

Lung transplantation is a salvage therapeutic option for patients with features of respiratory failure and those developing pulmonary hypertension. The prognosis of patients undergoing transplantation does not significantly differ from that of patients with other interstitial lung diseases. The 1-year survival rate is 75%; notably, >50% of patients survive for 5 years. Patients with multisystem LCH and lung involvement have a worse prognosis after transplantation, and recurrence of LCH in the transplanted organ has been found in approximately 20% of affected patients (33). Based on 87 transplant patients, Wajda et al. (81) reported outcomes similar to those of prior studies; 1-year, 5-year, and 10-year survival rates were 85, 49, and 22%, respectively. Moreover, the median survival was significantly better for women than for men (mean survival time, 9.3 vs. 3.9 years).

#### Follow-Up Examination

Patients with PLCH should undergo systematic follow-up examinations, initially performed after 3–4 months with subsequent evaluations at intervals of 3–12 months, depending on disease activity (7). HRCT is not necessary at every follow-up examination because it is not highly sensitive for evaluation of disease activity (34). Similarly, assessments of organs other than the lungs are not required; however, these evaluations should be performed in the event of new symptoms. Pulmonary function tests are important in follow-up assessment of patients with PLCH. Deterioration of exercise tolerance or the presence of new symptoms requires close examination. Participation in tobacco and marijuana cessation programs is highly recommended.

## Prognosis

The natural course and prognosis of PLCH are unpredictable and variable. Spontaneous regression and/or stabilization of the disease after smoking cessation, as well as the presence of addiction to substances other than tobacco in some patients, results in loss to follow-up for many patients; this may influence survival assessment. However, patients with PLCH were reported to exhibit lower mean survival compared to sex- and age-matched members of the general population (7). Vassallo et al. (9) observed a median survival of 12.5 years in adult patients with biopsy-proven PLCH.

Reduced spirometry parameters, low diffusing capacity of the lungs for carbon monoxide, severe cystic lesions detected on HRCT, low oxygen arterial pressure, older age, pulmonary hypertension, and multisystem LCH are negative prognostic factors. Moreover, a higher St. George's Respiratory Questionnaire (SGRQ) score and continued tobacco smoking have negative impacts on survival. Tazi et al. (16) presented an early decline of pulmonary function parameters (over 15%) in 40% of newly diagnosed PLCH patients, but in only 10% of them, progression of pulmonary lesions was noticed.

Recently, severe lung involvement in children was recognized as a factor that substantially influences the course of the disease and survival. Early administration of new targeted LCH therapies and viral infection prophylaxis are suggested (14).

Patients with PLCH also have a higher risk of infections; therefore, influenza and pneumococcal vaccines are recommended.

In addition, the presence of *BRAF*^V600E^ mutations and MS RO+ LCH in children was recognized as a factor that negatively influences prognosis (71), but in adults with PLCH, the *BRAF*^V600E^ mutation was not associated with LCH presentation and outcome (5).

LCH promotes the development of other neoplasms originating from the lymphatic and hematopoietic systems, including LCH overlapping with chronic myelogenous leukemia. In a group of 132 adult patients with LCH, Ma et al. (82) found that 32% were diagnosed with additional neoplasms, mostly before [58%] and concurrently (21%) with LCH diagnosis. Lung (18%), breast (18%), and colon (12%) cancers were the most frequently detected additional neoplasms.

### Pregnancy and Labor

Pregnancy does not worsen the course of PLCH, but delivery by cesarean section is recommended due to the enhanced likelihood of pneumothorax (83).

### Air Travel

Patients with PLCH have a higher risk of pneumothorax during air travel. However, the risk is not high; approximately 1% of patients exhibit this condition, and no severe instances of pneumothorax have been reported thus far (84).

## Conclusions

1. LCH is a rare disorder of unknown etiology caused by clonal proliferation of geno- and phenotypically altered LCs.
2. It usually involves the bones, lungs, skin and pituitary, lymphopoietic organs (lymph nodes, liver, spleen, bone marrow), gastrointestinal tract, thyroid, CNS, and others.
3. PLCH may be as follows: isolated, anticipating even for many years the occurrence of systemic changes, or from the very beginning, the lungs may be one of the sites involved.
4. Tobacco smoke is a crucial causative factor for PLCH. Smoking cessation is the most vital recommendation.
5. Chest HRCT has an outstanding importance in PLCH diagnosis. Pulmonary lesions are the following: centrilobular nodules, nodules with or without a central cavity, initially thick-walled cysts of various shapes that may convolute, forming the so-called clover leaves. The lesions are usually (>90%) sparing the costophrenic angles.
6. The definite diagnosis of PLCH is based on adequate clinical and radiological presentation and the identification of LCs in the examined tissue samples showing in the immunohistochemistry the presence of one of the following antigens: CD207 (langerin), CD1a.
7. Treatment of LCH depends on extension of the disease. Currently, the preferred cytostatic treatment option includes cladribine or cytarabine. Lung transplantation is recommended in patients with isolated PLCH with respiratory insufficiency.
8. PLCH patients have a lower mean survival than individuals of the same sex and age. Negative prognostic factors are age, tobacco smoking, severe obstruction, lowered PaO_2_, a higher score in SGRQ, pulmonary hypertension, and multiple organ involvement, particularly risk organs.

## Author Contributions

The author confirms being the sole contributor of this work and has approved it for publication.

## Conflict of Interest

The author declares that the research was conducted in the absence of any commercial or financial relationships that could be construed as a potential conflict of interest.

## Abbreviations

ARAF: A serine/threonine kinase
AKT: serine/threonine kinase
BRAF: B serine/threonine kinase
2-CDA: 2-chloro-2'-deoxyadenosine
CNS: central nervous system
ERK: extracellular signal-regulated kinase
HRCT: High-resolution computed tomography
LAM: lymphangioleiomyomatosis
LC: Langerhans cell
LCH: Langerhans cell histiocytosis
MAPK: mitogen-activated protein kinase
MS-LCH-: multisystem Langerhans cell histiocytosis
mTOR: mechanistic target of rapamycin
P: phosphorylation
PIK: phosphatidylinositol kinase
PIP-1: phosphatidylinositol monophosphate
PIP-2: phosphatidylinositol biphosphate
PLCH: pulmonary Langerhans cell histiocytosis
PTEN: phosphate and tensin homolog
SS-LCH: single system Langerhans cell histiocytosis.
